# Effect of Preoperative 2‐Hour Carbohydrate Load on Pediatric Patients Undergoing Elective Surgery: A Randomized Controlled Study

**DOI:** 10.1155/anrp/5580879

**Published:** 2025-12-03

**Authors:** Wafaa Madhy Atia Abdelwahed, Wafaa Mohamed Abdelelsalam, Ola Ahmed Taha, Shaimaa Abdelbadie, Amany Mohamed Abo Taleb

**Affiliations:** ^1^ Anesthesiology, Surgical Intensive Care and Pain Medicine Department, Faculty of Medicine, Tanta University, Tanta, Egypt, tanta.edu.eg; ^2^ Anesthesiology, Surgical Intensive Care and Pain Medicine Department, Faculty of Medicine, Kafr El-sheikh University, Kafr El-sheikh, Egypt, kfs.edu.eg; ^3^ Pediatrics Department, Faculty of Medicine, Tanta University, Tanta, Egypt, tanta.edu.eg; ^4^ Lecturer of Medical Microbiology and Immunology, Faculty of Medicine, Nahda University, Beni Suef, Egypt, nahdauniversity.org; ^5^ Anesthesiology, Surgical Intensive Care and Pain Medicine Department, Faculty of Medicine, Tanta University, Tanta, Egypt, tanta.edu.eg

**Keywords:** carbohydrate loading, inflammation, insulin resistance, pediatrics, personal satisfaction

## Abstract

**Background:**

Preoperative prolonged fasting can lead to metabolic disturbances and discomfort in pediatrics. This work aimed to assess the systemic effect of different preoperative carbohydrate (CHO) loads in pediatrics undergoing elective surgery.

**Methods:**

This randomized single‐blinded controlled trial was performed on 90 children, aged 5–10 years old, who were scheduled for elective surgery. Three equal groups were randomly assigned to patients. 2 h before surgery, patients received 10 mL/kg apple juice in Group 1, 1.75 mg/kg anhydrous glucose in Group 2, or water in Group 3. All patients followed standard fasting guidelines.

**Results:**

Inflammatory markers were notably elevated in Group 3 than in Groups 1 and 2 at the induction of anesthesia and 4 h after operation (*p* < 0.001). Insulin resistance (IR) markers significantly decreased in Group 3 at the induction of anesthesia but were significantly higher at 4 h after operation than in Groups 1 and 2 (*p* < 0.05). Random blood sugar was notably lower in Group 3 than in Groups 1 and 2 at induction of anesthesia and intraoperative but was significantly higher at 4 h after operation (*p* < 0.05). The patients’ parents were significantly more satisfied in Groups 1 and 2 in comparison to Group 3 (*p* = 0.003).

**Conclusions:**

In pediatrics undergoing elective surgeries, preoperative CHO loading is a safe alternative to standard fasting as it results in better preservation of IR markers, inflammatory response, and parents’ satisfaction levels.

**Trial Registration:**

ClinicalTrials.gov identifier: NCT06833671

## 1. Introduction

Children undergoing surgery experience stress due to disruptions in their daily routine and exposure to various perioperative circumstances that induce worry and discomfort [1]. Surgery itself is a type of physiological stress that triggers endocrine responses, whereas psychological stress involves feelings of anxiety and fear [2].

The management of perioperative nutrition in pediatric patients is a crucial aspect of patient care. Practically, patients have been advised to fast for several hours before surgery to avoid aspiration risk during the induction of anesthesia [3].

The current preoperative fasting guidelines for pediatric surgery are as follows: a 6‐hour fasting period for solid meals, formula milk, or cow milk, a 4‐hour fasting period for breast milk or unclear fluid, and a 2‐hour fasting period for clear fluids [4, 5].

Prolonged fasting results in metabolic disturbances, dehydration, and increased insulin resistance (IR) and is responsible for the notable role of postoperative nausea and vomiting. Additionally, the inflammatory response to surgery and other responses, such as postoperative pain, may be influenced [6].

In recent years, the concept of preoperative carbohydrate (CHO) loading has gained attention as a potential strategy to mitigate these drawbacks. The administration of a CHO‐rich drink 2 hours preoperative has been proposed to maintain euglycemia, decline IR, and enhance postoperative recovery [7, 8].

Preoperative infusion of CHOs could successfully reduce dehydration‐related adverse effects and IR, a main underlying etiology for catabolic state of surgical procedure. Further evaluations presented that preoperative oral CHO within 2 h prior to the surgical procedure not only can reduce operation related complications including thirst, hunger, headache, nausea, and vomiting but also has no adverse effects due to gastric content aspiration as the transit time of this solution is less than 2 hours [9].

The preoperative CHO loading approach is based on evidence that a short‐term CHO load can stimulate insulin secretion, thereby promoting glucose uptake and utilization by the body’s tissues, while minimizing aspiration risk through anesthesia induction [10].

The effect of preoperative 2‐hour CHO loading on pediatric patients is an area of active research [11]. Thus, this work aimed to evaluate the systemic effects of different preoperative CHO loads in pediatrics undergoing elective surgeries.

## 2. Patients and Methods

This prospective randomized single‐blind controlled trial was performed on 90 children.

Inclusion criteria were children aged 5–10 years old, with American Society of Anesthesiologists (ASA) physical status I and II, scheduled for elective surgery.

The research was conducted between April 2022 and September 2022, after authorization from Tanta University Hospital Ethical Committee (approval code ID: 35506/5/22), and the study was conducted in accordance with the Helsinki Declaration. The caregivers provided informed written consent.

Exclusion criteria were diabetes mellitus, IR, renal or hepatic insufficiency, and esophageal or gastric surgery or gastrointestinal disorders (including gastroesophageal reflux, hiatal hernia, or gastritis).

### 2.1. Randomization and Blinding

Random allocation was conducted using computer‐generated randomization numbers, and the codes of each patient were kept in an opaque, sealed envelope. In a parallel manner, patients were randomized into three equal groups with a 1:1:1 allocation ratio.

A preoperative CHO load was administered to Group 1 with a commercial brand of apple juice (glucose 28 g in 250 mL) 2 hours before the operation. The dosage was 10 mL/kg, with a maximal volume of 250 mL. A preoperative CHO load was administered to Group 2 with anhydrous glucose (Alpha Chemika) 2 h before the operation. The dosage was 1.75 mg/kg/dose.

Group 3 received water 2 h before the operation. All patients followed standard fasting guidelines: a minimum of 2 h for clear liquids, such as water; 4 h for breast milk; 6 h for non‐human milk, infants′ formula, and light meals; and 8 h for solid foods. The doctors who assessed the study outcomes were blind to the group allocation.

Not all patients receive any premedication. On the day of surgery, each patient had two visits from the same doctor, once before and once after 6 h of fasting (2 hours before the surgery) for measuring random blood sugar (RBS) and assessment of signs of dehydration. On arrival at the operating room, immediately before general anesthesia was induced, the second assessment of RBS and dehydration signs was done.

C‐reactive protein (CRP), procalcitonin, neutrophil/lymphocyte ratio (NLR), insulin‐resistant markers, homeostatic model assessment for IR (HOMA‐IR), and c‐peptide were assessed 2 h before surgery as baseline, at induction of anesthesia, and 4 h after the operation.

An intravenous (IV) cannula was placed before anesthesia induction. Every patient received a standardized general anesthetic regimen consisting of 2 mg/kg propofol induction, 1 μg/kg fentanyl, and 0.5 mg/kg atracurium neuromuscular block. Maintenance was done with sevoflurane and an air‐oxygen mixture.

Any complications, such as perioperative nausea, vomiting, and aspiration, were documented.

Ringer lactate solution was used for intraoperative and postoperative fluid replacement in all groups. To ensure adequate hydration in parenteral fluid therapy, the 4/2/1 principle has been widely adopted, which calculates the maintenance requirement for water based on a patient’s weight. According to this principle, patients weighing less than 10 kg require 4 mL/kg, while those weighing between 10 and 20 kg require 40 mL plus 2 mL/kg for each additional 10 kg, and those weighing more than 20 kg require 60 mL plus 1 mL/kg for each additional 20 kg. Furthermore, the concept of third space loss has been recognized, with estimated rates of approximately 2 mL/kg/h for superficial surgery, 4–7 mL/kg/h for moderate incisions, and 5–10 mL/kg/h for large incisions. Intraoperative blood losses were replaced with isotonic solution or blood, contingent upon the patient’s hematocrit level [12].

The primary outcome was CRP level. The secondary outcomes were NLR, procalcitonin, and IR markers (insulin, HOMA‐IR, and C‐peptide), RBS, patients’ parents’ satisfaction, and complications.

### 2.2. Sample Size

The G∗Power 3.1.9.2 software (Universitat Kiel, Germany) was employed to determine the sample size. A pilot study was conducted, involving 5 cases per group, which revealed that the mean (± standard deviation [SD]) CRP levels at 4 h postoperation were 10.52 ± 0.72 mg/dL in Group 1, 10.66 ± 0.94 mg/dL in Group 2, and 11.3 ± 0.5 mg/dL in Group 3. The determination of the sample size was based on the following parameters: an effect size of 0.99, a 95% confidence level, a study power of 95%, a group ratio of 1:1:1, and an additional three cases per group to account for potential dropouts. Consequently, each group recruited a total of 30 patients.

### 2.3. Statistical Analysis

SPSS v27 (IBM©, Chicago, IL, USA) was employed to perform statistical analysis. The Shapiro–Wilk test and histograms were employed to assess the normality of the data distribution. The ANOVA (F) test with post hoc test (Tukey) was employed to analyze quantitative parametric data, which were presented as mean and SD. The chi‐square test was employed to analyze qualitative variables, which were presented as frequency and percentage. A two‐tailed *P* value that was less than 0.05 was considered statistically significant.

## 3. Results

**Figure 1:**
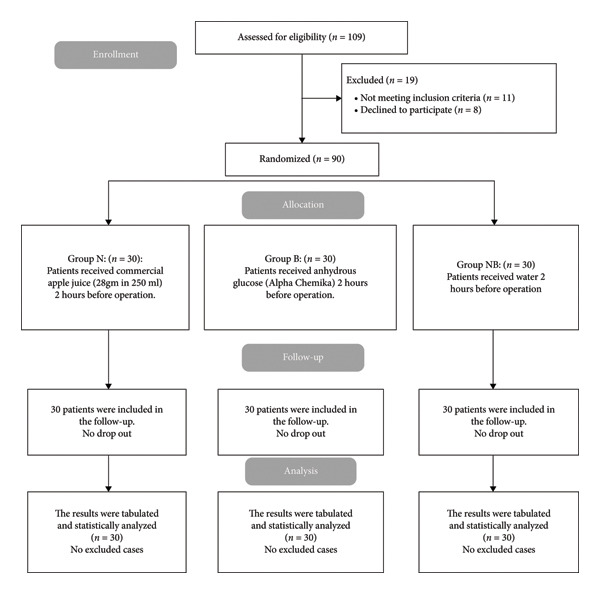
CONSORT flowchart of the enrolled patients.

One hundred nine patients were evaluated for eligibility in this study; 11 patients did not meet the criteria, and 8 patients’ guardians declined to participate. The remaining patients were randomly assigned to three equal groups, each containing 30 patients. The statistical analysis and follow‐up of all allocated patients were conducted (Figure 1).

Demographic data and duration of surgery were not significantly different among the three groups (Table 1).

**Table 1:** Demographic data and duration of surgery of the studied groups.

		Group 1 (*n* = 30)	Group 2 (*n* = 30)	Group 3 (*n* = 30)	*p* value
Age (years)		7.1 ± 1.59	6.9 ± 1.09	7.4 ± 1.35	0.368
Sex	Male	17 (56.67%)	21 (70%)	19 (63.33%)	0.563
	Female	13 (43.33%)	9 (30%)	11 (36.67%)	
Weight (kg)		27.1 ± 6.41	26.6 ± 5.14	28.1 ± 5.79	0.573
ASA physical status	I	20 (66.67%)	24 (80%)	23 (76.67%)	0.468
	II	10 (33.33%)	6 (20%)	7 (23.33%)	
Duration of surgery (min)		55.3 ± 15.14	62.8 ± 16.22	57.2 ± 18.18	0.194

*Note:* Data are presented as mean ± SD or frequency (%).

Abbreviation: ASA, American Society of Anesthesiologists.

CRP, NLR, and procalcitonin were comparable at baseline among the three groups. CRP, NLR, and procalcitonin were comparable between Groups 1 and 2 at induction of anesthesia and at 4 h after operation and were significantly elevated in Group 3 than Groups 1 and 2 at induction of anesthesia and 4 h after operation (*p* < 0.001) (Table 2).

**Table 2:** Inflammatory markers of the studied groups.

		Group 1 (*n* = 30)	Group 2 (*n* = 30)	Group 3 (*n* = 30)	*P*	Post hoc
CRP (mg/dL)	Baseline	1.96 ± 1.49	1.87 ± 1.4	2.13 ± 1.22	0.753	
	At the induction of anesthesia	4.37 ± 1.48	4.74 ± 1.38	8.06 ± 1.51	< 0.001^∗^	*P*1 = 0.590
						*P*2 < 0.001^∗^
						*P*3 < 0.001^∗^
	4 h after the operation	7.95 ± 1.66	8.39 ± 1.33	14.28 ± 3.81	< 0.001^∗^	*P*1 = 0.781
						*P*2 < 0.001^∗^
						*P*3 < 0.001^∗^

NLR	Baseline	1.27 ± 0.23	1.35 ± 0.28	1.4 ± 0.32	0.188	
	At the induction of anesthesia	1.44 ± 0.24	1.62 ± 0.34	2 ± 0.4	< 0.001^∗^	P1 = 0.090
						P2 < 0.001^∗^
						P3 < 0.001^∗^
	4 h after the operation	1.61 ± 0.28	1.74 ± 0.35	2.53 ± 0.36	< 0.001^∗^	P1 = 0.241
						P2 < 0.001^∗^
						P3 < 0.001^∗^

Procalcitonin (ng/mL)	Baseline	0.29 ± 0.09	0.28 ± 0.06	0.31 ± 0.09	0.282	
	At the induction of anesthesia	0.87 ± 0.1	0.93 ± 0.09	1.1 ± 0.12	< 0.001^∗^	P1 = 0.068
						P2 < 0.001^∗^
						P3 < 0.001^∗^
	4 h after the operation	1.04 ± 0.1	1.13 ± 0.09	1.44 ± 0.28	< 0.001^∗^	P1 = 0.147
						P2 < 0.001^∗^
						P3 < 0.001^∗^

*Note:* Data are presented as mean ± SD. P1, *p* value between Group 1 and Group 2; P2, *p* value between Group 1 and Group 3; P3, *p* value between Group 2 and Group 3.

Abbreviations: CRP, C‐reactive protein; NLR, neutrophil‐to‐lymphocyte ratio.

^∗^Significant difference as *p* value ≤ 0.05.

Insulin, HOMA‐IR, and C‐peptide were comparable at baseline among the three groups: insulin, HOMA‐IR, and C‐peptide were comparable at induction of anesthesia and 4 h after operation anesthesia between Groups 1 and 2 and were significantly decreased in Group 3 than Groups 1 and 2 at induction of anesthesia (*p* < 0.05) and were notably elevated in Group 3 than Groups 1 and 2 at 4 h after operation (*p* < 0.05) (Table 3).

**Table 3:** Insulin, HOMA.IR, C‐peptide, and random blood sugar of the studied groups.

		Group 1 (*n* = 30)	Group 2 (*n* = 30)	Group 3(*n* = 30)	*p* value	Post hoc
Insulin (μU/mL)	Baseline	0.33 ± 0.12	0.34 ± 0.16	0.27 ± 0.12	0.133	
	At the induction of anesthesia	1.1 ± 0.22	1.05 ± 0.28	0.32 ± 0.14	< 0.001^∗^	*P*1 = 0.651
						*P*2 < 0.001^∗^
						*P*3 < 0.001^∗^
	4 h after the operation	0.44 ± 0.16	0.48 ± 0.17	0.60 ± 0.14	< 0.001^∗^	*P*1 = 0.538
						*P*2 < 0.001^∗^
						*P*3 = 0.011^∗^
HOMA.IR	Baseline	1.25 ± 0.28	1.23 ± 0.26	1.33 ± 0.32	0.361	
	At the induction of anesthesia	2.36 ± 0.36	2.25 ± 0.3	1.43 ± 0.32	< 0.001^∗^	P1 = 0.421
						P2 < 0.001^∗^
						P3 < 0.001^∗^
	4 h after the operation	1.45 ± 0.29	1.46 ± 0.27	2.18 ± 0.37	< 0.001^∗^	P1 = 0.985
						P2 < 0.001^∗^
						P3 < 0.001^∗^
C‐peptide (ng/mL)	Baseline	1.28 ± 0.27	1.3 ± 0.3	1.25 ± 0.3	0.781	
	At the induction of anesthesia	1.47 ± 0.26	1.49 ± 0.31	1.28 ± 0.31	0.01^∗^	P1 = 0.963
						P2 = 0.033^∗^
						P3 = 0.016^∗^
	4 h after the operation	1.3 ± 0.27	1.33 ± 0.3	1.59 ± 0.31	< 0.001^∗^	P1 = 0.939
						P2 < 0.001^∗^
						P3 = 0.003^∗^
Random blood sugar	Baseline	96 ± 13.24	98.2 ± 12.02	95.7 ± 12.6	0.705	
	At the induction of anesthesia	107.27 ± 13.44	104.07 ± 12.33	96.17 ± 12.69	0.004^∗^	P1 = 0.600
						P2 = 0.003^∗^
						P3 = 0.05^∗^
	Intraoperative	108.13 ± 13.55	105.1 ± 12.93	96.97 ± 12.63	0.004^∗^	P1 = 0.641
						P2 = 0.004^∗^
						P3 = 0.046^∗^
	After recovery	100.4 ± 13.49	94.47 ± 9.71	98.57 ± 12.66	0.155	
	4 h after the operation	97.43 ± 13.71	95.23 ± 9.32	106.2 ± 12.76	0.002^∗^	P1 = 0.761
						P2 = 0.017^∗^
						P3 = 0.002^∗^

*Note:* Data are presented as mean ± SD. P1, *p* value between Group 1 and Group 2; P2, *p* value between Group 1 and Group 3; P3, *p* value between Group 2 and Group 3.

Abbreviation: HOMA.IR, homeostatic model assessment for insulin resistance.

^∗^Significant difference as *p* value ≤ 0.05.

RBS was insignificantly different at baseline and after recovery among the three groups and was comparable at induction of anesthesia, intraoperative, and 4 h after operation between Groups 1 and 2. RBS was notably decreased in Group 3 than Groups 1 and 2 at induction of anesthesia and intraoperative and was notably elevated in Group 3 than Groups 1 and 2 at 4 h after operation (*p* < 0.05) (Table 3).

The patients’ parents were significantly more satisfied in Groups 1 and 2 in comparison to Group 3 (*p* = 0.003). Nausea and vomiting were comparable among the three groups. Aspiration did not occur in any patient of the three groups (Table 4).

**Table 4:** Parents’ satisfaction and complications of the studied groups.

		Group 1 (*n* = 30)	Group 2 (*n* = 30)	Group 3 (*n* = 30)	*p* value
Parents’ satisfaction	Satisfied	25 (83.33%)	15 (50%)	10 (33.33%)	0.003^∗^
	Neutral	4 (13.33%)	10 (33.33%)	11 (36.67%)	
	Unsatisfied	1 (3.33%)	5 (16.67%)	9 (30%)	
Nausea		2 (6.67%)	3 (10%)	5 (16.67%)	0.455
Vomiting		1 (3.33%)	1 (3.33%)	3 (10%)	0.429
Aspiration		0 (0%)	0 (0%)	0 (0%)	—

*Note:* Data are presented as a frequency (%). P1, *p* value between Group 1 and Group 2; P2, *p* value between Group 1 and Group 3; P3, *p* value between Group 2 and Group 3.

^∗^Significant difference as *p* value ≤ 0.05.

## 4. Discussion

Children have higher metabolic rates and glucose utilization compared to adults, making them more prone to the negative impacts of prolonged fasting [13]. The stress response to surgery can be more pronounced in children, leading to greater IR and catabolism. These adverse effects can be mitigated by the oral intake of CHO, which attenuates postoperative IR and exists as a well‐defined methodology in adult patients [14]. Recent evidence highlights the importance of involving caregivers and patients as active participants in perioperative decision making, which may mitigate stress and improve compliance with preoperative protocols. For instance, Ursoleo et al. [15] demonstrated that structured engagement of families in perioperative planning enhances psychological preparedness and reduces physiological stress markers.

This study is the first to assess the systemic impacts of standard fasting and varying preoperative CHO intakes in pediatrics undergoing elective surgery in the existing literature.

In our study, inflammatory marker levels at induction of anesthesia and 4 h after the operation were notably elevated in the standard fasting group compared with CHO loading groups. However, the inflammatory marker levels were comparable between the two CHO loading groups.

The inflammatory response is a crucial aspect of the surgical stress response, and the higher inflammatory marker levels observed in the standard fasting group may be attributed to the exacerbated stress response due to the prolonged fasting state [16].

In agreement with our results, Haider and Ahmed [17] performed a meta‐analysis to assess the impact of preoperative CHO load on postoperative inflammation. They stated that CHO load reduced postoperative CRP and interleukin‐6 levels.

Also, Hu et al. [18] demonstrated that inflammatory marker (tumor necrosis factor and interleukin 6 and 8) levels on the first day postsurgery were notably elevated in the standard fasting group than in the CHO loading groups.

In the present study, IR markers and RBS levels at induction of anesthesia and 4 h after the operation were notably decreased in the standard fasting group in comparison to the CHO loading groups, but their levels were comparable between the two CHO loading groups.

Preoperative fasting lowers glucose levels due to the depletion of glucose reserves, resulting in reduced insulin secretion and lower C‐peptide levels. This decrease in insulin secretion also leads to a decrease in IR. CHO loading before surgery results in higher glucose levels due to the ingestion of CHO, leading to increased insulin secretion and higher C‐peptide levels. However, this increase in insulin levels can also lead to increased IR [19].

In accordance with our findings, Shi et al. [20] performed a meta‐analysis that included nine studies on 1211 patients. They noted that compared to the control group, HOMA‐IR notably decreased in the CHO group.

Also, Larid et al. [3] revealed that glucose levels in the blood were elevated in the CHO load group than in the placebo group.

Also, Lee et al. [21] stated that the standard fasting group exhibited significantly reduced insulin sensitivity in adult patients undergoing off‐pump coronary artery bypass compared to the CHO loading group.

Additionally, Tamura et al. [22] showed that oral CHO‐rich drinks can reduce IR and metabolic stress.

In this study, the patients’ parents were significantly more satisfied in CHO groups than in standard fasting groups. Nausea and vomiting incidence were higher in the standard fasting group than CHO group. Satisfaction among children improved in the CHO group, particularly regarding agitation, anger, and crying.

In accordance with our findings, Shi et al. [20] showed that during cesarean section, nausea and vomiting frequency was reduced by preoperative oral CHO.

Tudor‐Drobjewski et al. [23] revealed that nausea and vomiting incidence was higher in the CHO loading group compared to the standard fasting group.

Nevertheless, Doo e al. [24] concluded that preoperative oral CHO did not seem to have any significant impact on patient satisfaction compared to the standard fasting group before thyroidectomy. This difference may be attributed to the difference in age between adults.

Beyond nutritional interventions, emerging anesthetic agents such as remimazolam (a short‐acting benzodiazepine) show promise in accelerating postoperative recovery. Its rapid onset and short half‐life [25] enable swift emergence from anesthesia, facilitating early airway patency and feeding resumption. In both adult and pediatric populations, intranasal or IV remimazolam administration has been associated with reduced recovery times and improved hemodynamic stability [26, 27]. While our study focused on preoperative CHO loading, combining such pharmacological adjuvants with metabolic optimization strategies could synergistically enhance recovery pathways, particularly in high‐stress pediatric cohorts.

Our findings align with principles of enhanced recovery after surgery (ERAS) protocols, which advocate minimizing metabolic stress and accelerating postoperative recovery. Monaco et al. [28] recently outlined a pediatric ERAS pathway for catheterization procedures, emphasizing multimodal analgesia and early feeding. Specifically, we compared our findings with their recommendations, highlighting similarities in reducing postoperative nausea and vomiting and differences in the use of intraoperative fluids. Future studies could integrate both strategies to evaluate compounded benefits in pediatric surgical care.

The findings’ generalizability to other patient populations or surgical contexts may be restricted by the fact that the study population was composed of pediatric patients who underwent elective surgery. Further multicenter studies with large sample sizes are recommended.

## 5. Conclusions

In pediatric patients undergoing elective surgery, preoperative CHO loading, either by apple juice and anhydrous glucose, is a safe and satisfactory substitute for standard fasting, as it results in better preservation of IR markers, lower inflammatory response, and higher parent satisfaction levels compared to standard fasting.

## Ethics Statement

The research was conducted between April 2022 and September 2022, after authorization from Tanta University Hospital Ethical Committee (approval code ID: 35506/5/22), and the study was conducted in accordance with the Helsinki Declaration. The caregivers provided informed written consent.

## Conflicts of Interest

The authors declare no conflicts of interest.

## Author Contributions

Study concept and design: W.M.A.A., A.M.A.T., and W.M.A. Analysis and interpretation of data: O.A.T. Drafting of the manuscript: S.A. Critical revision of the manuscript for important intellectual content: W.M.A. A., A.M.A.T., and W.M.A.

## Funding

No funding was received for conducting this study.

## Data Availability

Data are available on reasonable request from the corresponding author.
